# Reply to: Camptocormia due to myotinilopathy, Parkinson’s disease, or both?

**DOI:** 10.1186/s42466-023-00284-2

**Published:** 2023-09-27

**Authors:** Jan Niklas Petry-Schmelzer, Gilbert Wunderlich

**Affiliations:** 1 Faculty of Medicine and University Hospital, Department of Neurology, University of Cologne, Cologne, Germany; 2 Faculty of Medicine and University Hospital, Center for Rare Diseases, University of Cologne, Cologne, Germany

## Supplement: none

We thank the author for his interest in our case report “Myofibrillar myopathy – a rare but important differential diagnosis of camptocormia in a patient with Parkinson’s Disease” [1]. First of all, we agree that the aim of this case report was to highlight the importance of a careful history taking and neurological examination in patients presenting with camptocormia as concurrent pathomechanisms might be present. However, as already indicated in the title, we concluded that myofibrillar myopathy is a rare differential diagnosis of camptocormia in patients with Parkinson’s Disease and not vice versa. In our opinion, resolving this misinterpretation already answers most of the raised questions regarding the patient’s history, and we thank the author for highlighting again the importance of the order of symptom development as a key to the correct diagnosis. As presented in the supplemental video, upper-limb weakness was clinically evident, but unfortunately the timepoint of the onset of upper-limb weakness was not reported by the patient, as she was not aware of it at the initial presentation. We agree with the author, that fatty atrophy might also have been present in other body regions, but as mentioned in the study, MRI was only available for paraspinal muscles. We also agree that the creatine kinase is an important marker in the evaluation of patients with suspected myopathy but following the mentioned case-series by Selcen et al. [2] it is not uncommon that creatine kinase levels are normal in myofibrillar myopathy and other myopathies [3]. As highlighted by the author and in our discussion, it is mandatory to screen patients with myotilinopathy for cardiac involvement. We received the results at the follow-up after submission of the manuscript and so far, there is no evidence of cardiac involvement in our patient. Lastly, we thank the author for the interesting note that postural abnormalities might even be observed in up to 27% of patients with Parkinson’s Disease. However, we would like to emphasize that the reported prevalence includes all kinds of postural abnormalities and not solely camptocormia [4]. But this once more highlights the importance of a thorough clinical workup of patients presenting with camptocormia.
